# Supplementary material for: Spin selection in atomic-level chiral metal oxide for photocatalysis
Source: Nat Commun. 2023 Jul 28;14:4562. doi: 10.1038/s41467-023-40367-x (PMC10382512; doi:10.1038/s41467-023-40367-x)
Supplement: Supplementary file 1 — Supplementary Information [file 41467_2023_40367_MOESM1_ESM.pdf]

Supplementary Information (SI)

Spin selection in atomic-level chiral metal oxide for photocatalysis

Minhua Ai[1,2], Lun Pan[1,2,3*], Chengxiang Shi[1,2,3], Zhen-Feng Huang[1,2,3], Xiangwen Zhang[1,2,3], Wenbo Mi[4], Ji-Jun Zou[1,2,3*]

[1]Key Laboratory for Green Chemical Technology of the Ministry of Education, School of Chemical Engineering and Technology, Tianjin University, Tianjin 300072, China.

[2]Collaborative Innovative Center of Chemical Science and Engineering (Tianjin), Tianjin 300072, China.

[3]Haihe Laboratory of Sustainable Chemical Transformations, Tianjin, 300192, China.

[4]Tianjin Key Laboratory of Low Dimensional Materials Physics and Preparation Technology, School of Science, Tianjin University, Tianjin 300354, China.

[*] E-mail: panlun76@tju.edu.cn (L. Pan); jj_zou@tju.edu.cn (J.-J. Zou).

1. Supplementary Methods

Materials: L/D/DL-methionine were purchased from Meryer Chemical Technology Co., Ltd; zinc acetate and potassium permanganate ($KMnO_4$) were from Tianjin Guangfu Fine Chemical Research Institute; n-butanol was from Tianjin Kemiou Chemical Reagent Co., Ltd; RhB was from Aladdin Industry Corporation. The ultrapure water with a resistivity higher than 18.2 MΩ·cm was used in all experiments.

Substrate activation: Fluorine-doped tin oxide (FTO) glass substrates were cleaned ultrasonically with acetone, ethanol and water in sequence. Then, the FTO glass was immersed in 20 mL of a 10 mM fresh $KMnO_4$ aqueous solution with 50 μL of *n*-butanol as the reducing agent and kept at 85 ^{o}C for 20 min. After that, the activated substrates were rinsed with water and ethanol and dried at 60 ^{o}C.

General structure characterizations: X-ray diffraction (XRD) characterization were recorded at a scanning rate of 7 ^{o}/min under Cu Kα radiation at 40 kV and 40 mA (Bruker, D8-Focus). Scanning electron microscope (SEM) images were observed using a field-emission scanning electron microscope (Hitachi, Regulus 8100). Transmission electron microscopy (TEM) and elemental mapping were carried out using a field emission transmission electron microscope (JEOL, JEM-F 200 and 2100). X-ray photoelectron spectrum (XPS) analysis was conducted with a PHI-1600 XPS system equipped with Al Kα radiation, and the C1s peak (284.8 eV) was calibrated as contamination carbon. UV-vis diffuse reflectance spectra (UV-vis DRS) were obtained by using $BaSO_4$ as reference (Shimadzu, UV-2600). Thermogravitity (TG) analysis was conducted on a TGA Q500 thermogravimeter under an air atmosphere with a rate of 5 °Cmin^{-1}. The specific rotation of chiral ZnO photoandodes were measured by a polarimeter (Germany, KRÜSS P8000) with the specific rotation of 0.29° and -0.84° under the test wavelength of 589 nm.

2. Supplementary Figures

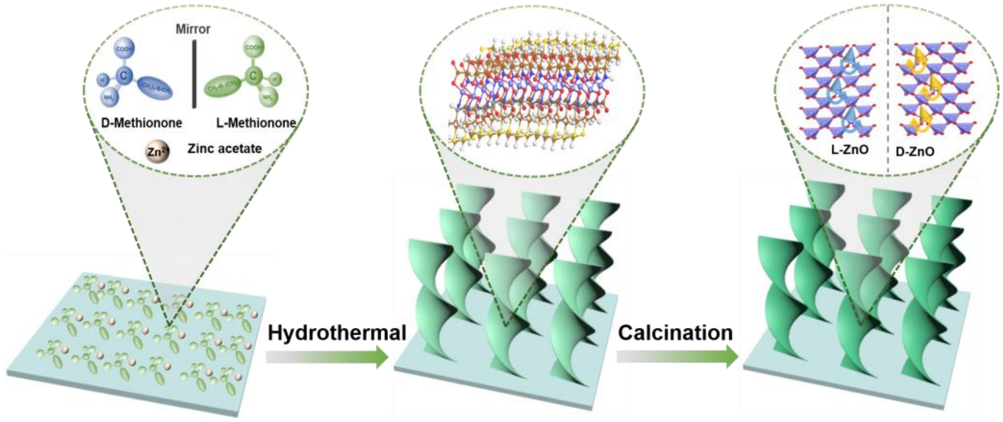

Figure S1. Schematic diagram of chiral film fabrication.

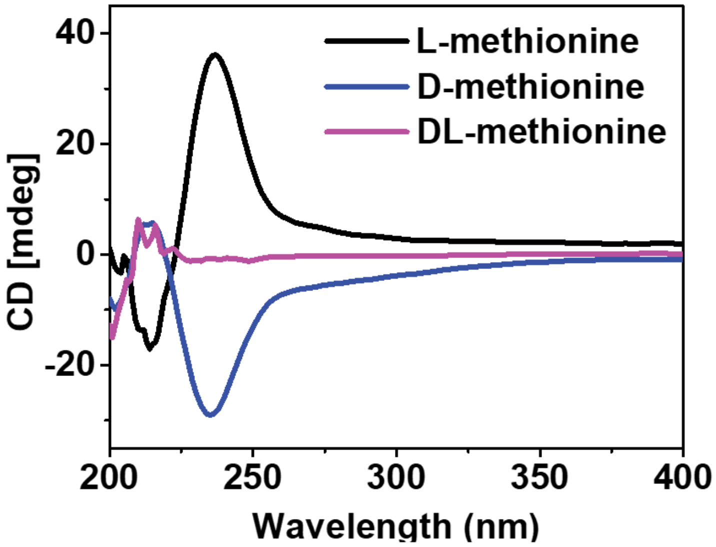

Figure S2. TCD spectra of solid L-, D- and DL-methionine.

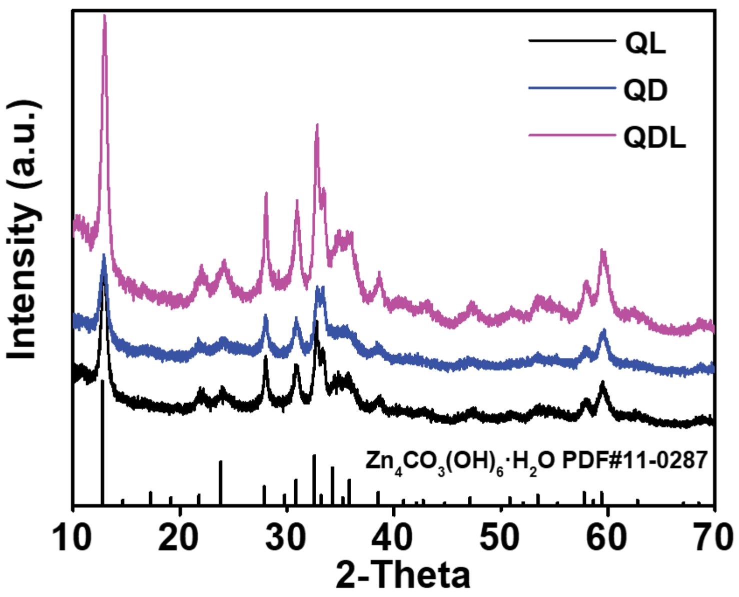

Figure S3. X-ray diffraction patterns of zinc carbonate hydroxide hydrate synthesized by hydrothermal processes. QL-ZnO, QD-ZnO and QDL-ZnO are the precursors of L-, D- and DL-ZnO, respectively.

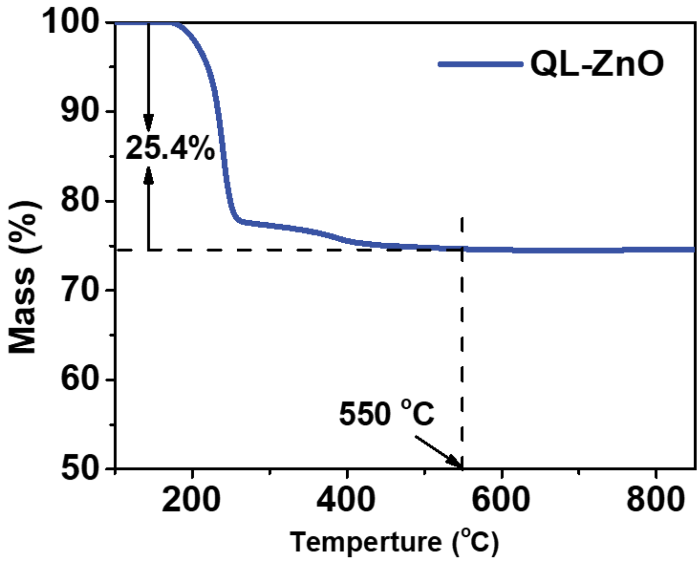

Figure S4. TG curves of QL-ZnO. QDL-ZnO is the precursor of DL-ZnO.

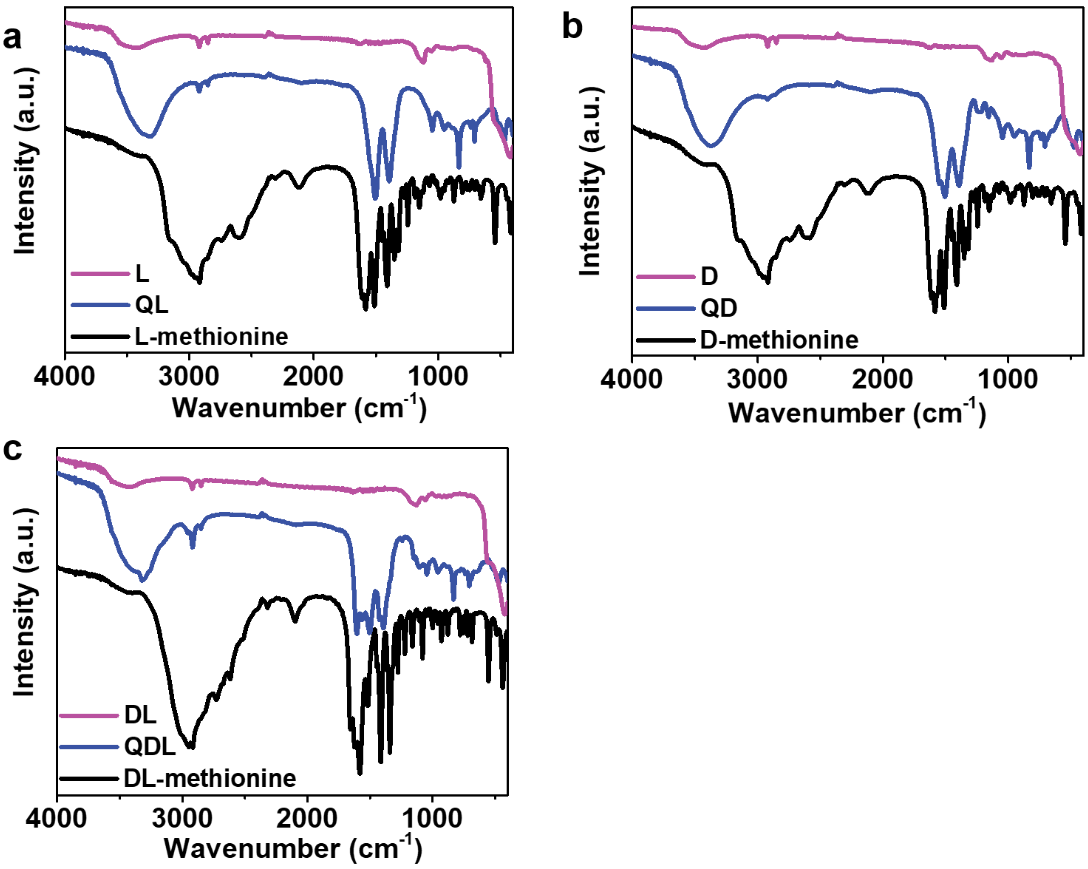

Figure S5. FTIR spectra of (a) L-methionine (black line), QL-ZnO (blue line) and L-ZnO (purple line); (b) D-methionine (black line), QD-ZnO (blue line) and D-ZnO (purple line); (c) DL-methionine(black line), QDL-ZnO (blue line) and DL-ZnO (purple line). QL-ZnO, QD-ZnO and QDL-ZnO is precursor of L-, D- and DL-ZnO, respectively.

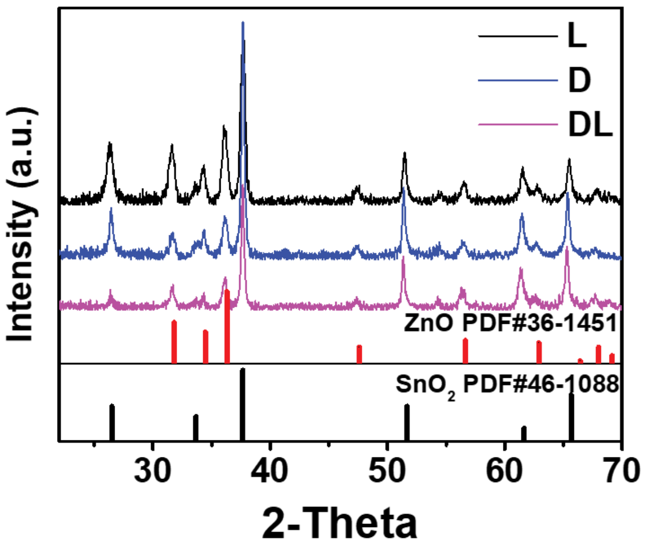

Figure S6. XRD patterns of L-ZnO, D-ZnO and DL-ZnO.

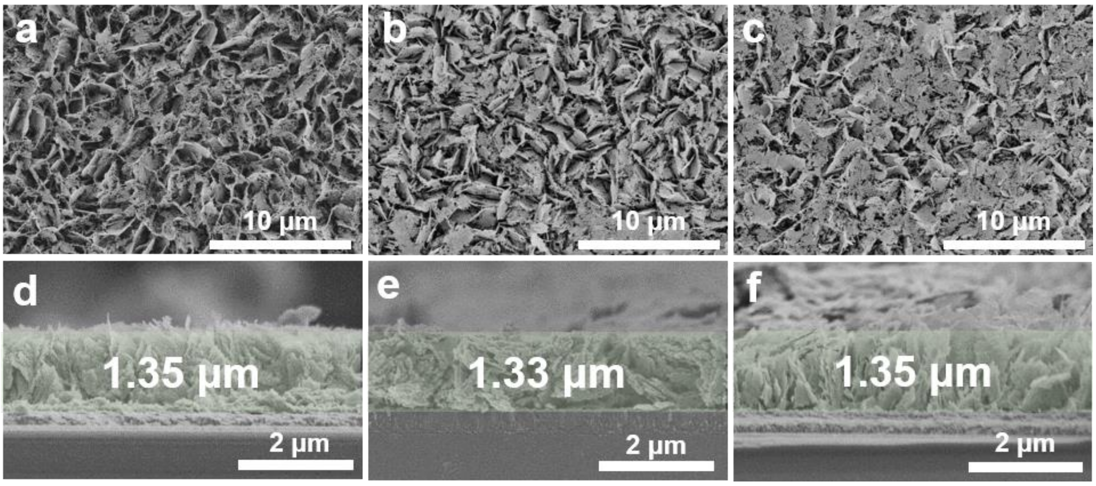

Figure S7. Top-view and cross-sectional SEM images of (a, d) L-ZnO, (b, e) D-ZnO and (c, f) DL-ZnO.

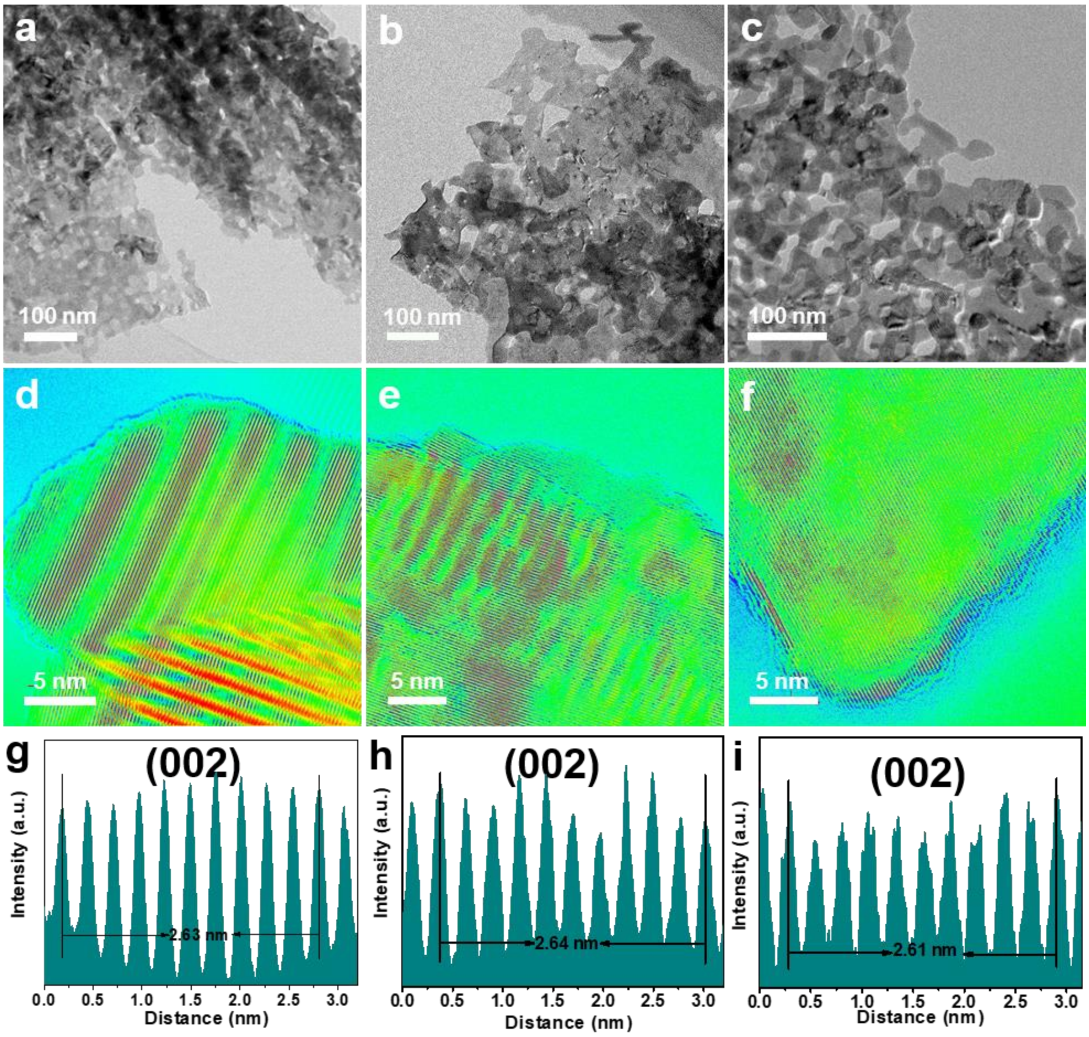

Figure S8. TEM images of (a) L-ZnO, (b) D-ZnO and (c) DL-ZnO. HRTEM images and the corresponding lattice fringe spacing of (d) and (g) L-ZnO, (e) and (h) D-ZnO, (f) and (i) DL-ZnO.

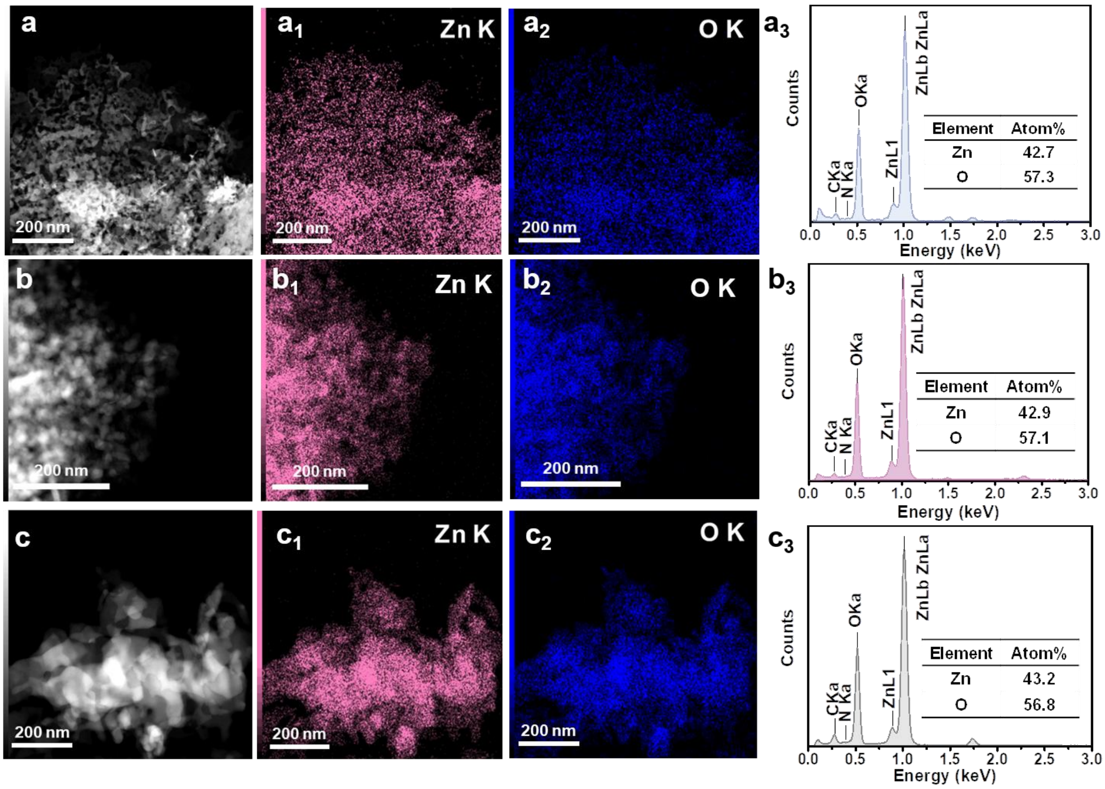

Figure S9. Energy-dispersive X-ray spectroscopy (EDS) of (a) L-ZnO, (b) D-ZnO and (c) DL-ZnO.

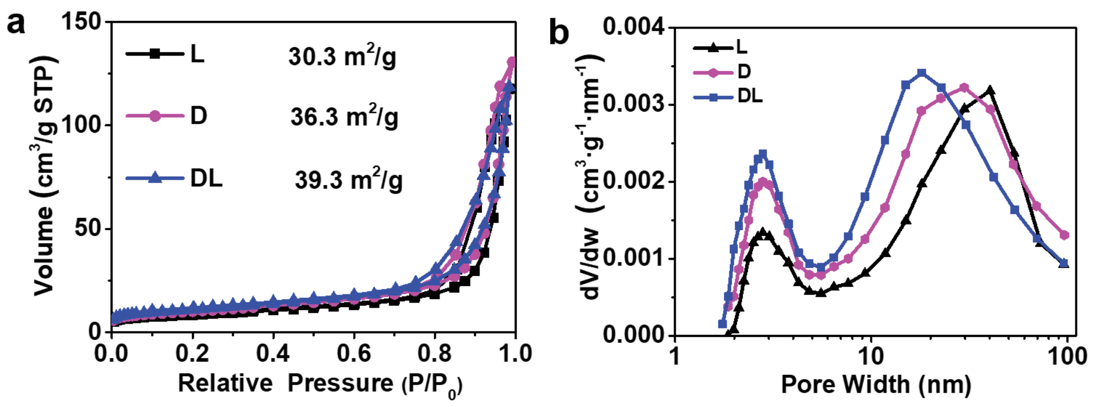

Figure S10. Specific surface area and pore structure. (a) N_2 adsorption-desorption isotherm curves and (b) Pore size distribution of L-ZnO, D-ZnO and DL-ZnO.

The surface area and porosity structure have important effects on the light absorption and activity of photocatalysts[1-3], which were investigated by N_2 adsorption-desorption measurements (Figure S10, SI). All samples show typical IV isotherms with H_3-type hysteresis loops, indicating the presence of mesopores. The BET surface area values of L-ZnO, D-ZnO and DL-ZnO are 30.3, 36.3 and 39.3 cm^2/g, respectively. Moreover, L-ZnO, D-ZnO and DL-ZnO possess the similar mesopores with size of 2.8 nm, and they also have mesopores with size of 40.1, 29.8 and 18.3 nm, respectively. The total pore volumes of L-ZnO, D-ZnO and DL-ZnO are 0.18, 0.20, and 0.18 cm^3/g, respectively. Although the presence of mesoporous structure is beneficial to enhance light scattering/reflection and provide active sites, the photocatalytic activity difference between the three samples due only to the surface area and porosity structure will be relatively small.

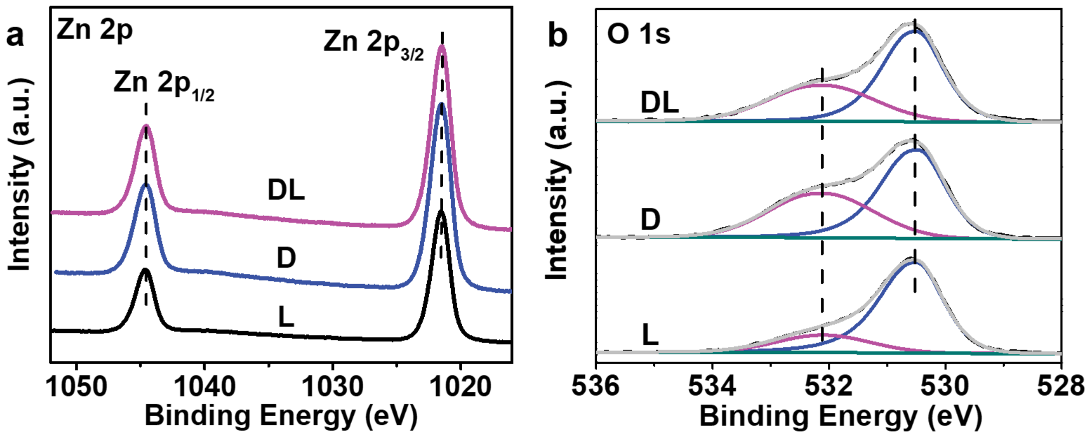

Figure S11. High-resolution XPS of (a) Zn 2p and (b) O1s of L-ZnO, D-ZnO and DL-ZnO.

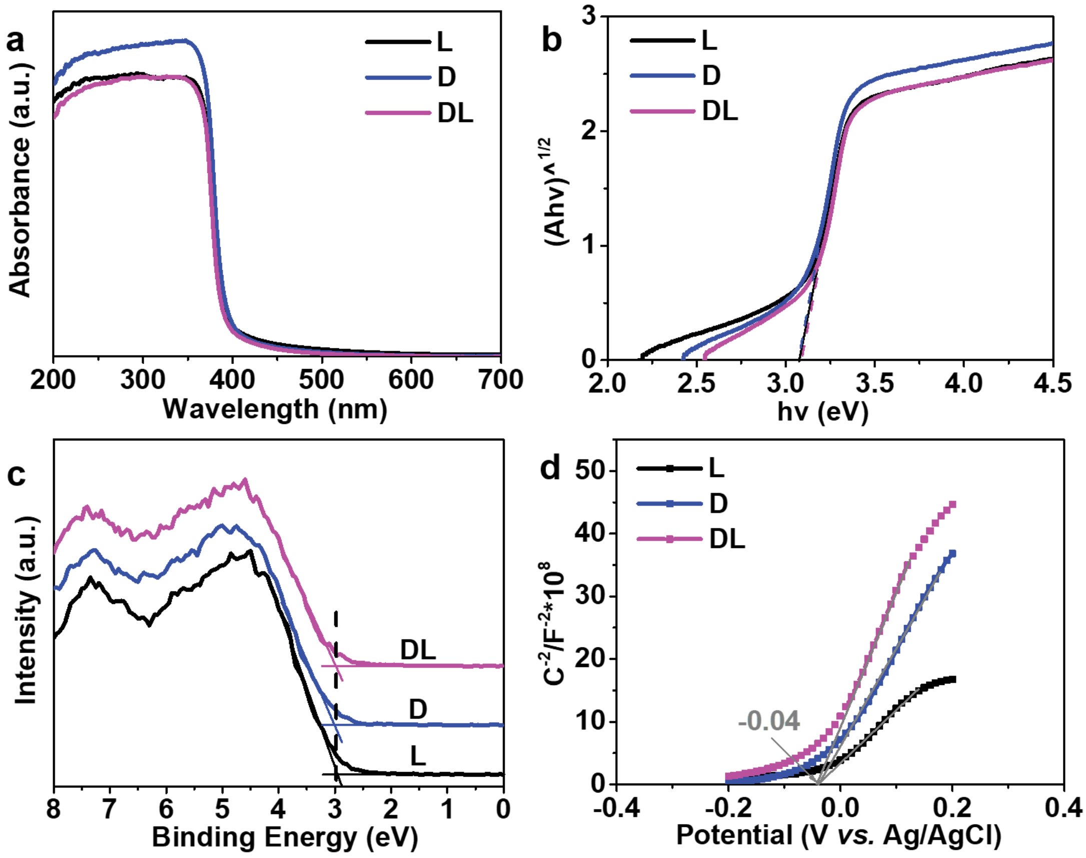

Figure S12. Optical properties and band structures. (a) UV–vis absorption spectra; (b) calculated band gap; (c) XPS valence band; (d) MS polts of L-ZnO, D-ZnO and DL-ZnO.

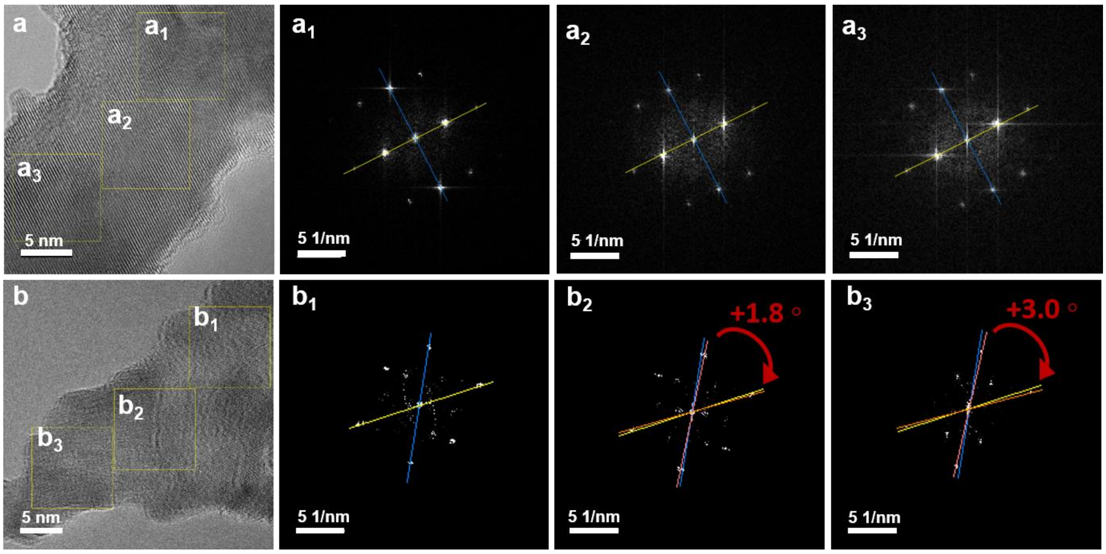

Figure S13. (a) HRTEM image and (a_1-a_3) the corresponding Fast Fourier transform images of DL-ZnO nanoplates. (b) HRTEM image and (b_1-b_3) the corresponding Fast Fourier transform images of D-ZnO.

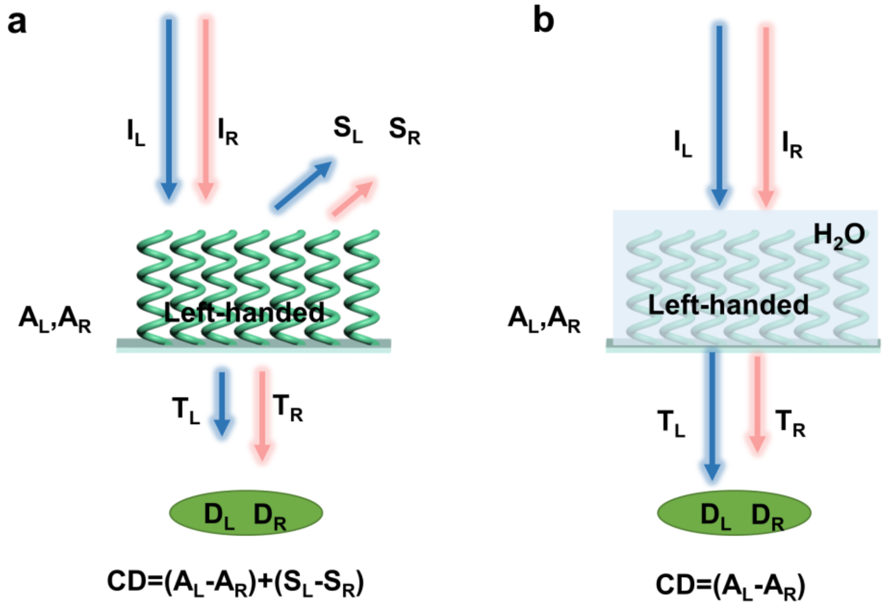

Figure S14. Schematic illustration of transmitted CD spectra of (a) the sample and (b) the sample infiltrated with water (isotropic liquid). Red and blue arrows represent to right- and left-handed polarized light, respectively. I is the incident light (I_L=I_R), A is the absorbed light, T is the transmitted light, S is the scattered light, D is the detected light.[4] Copyright 2022 Wiley-VCH.

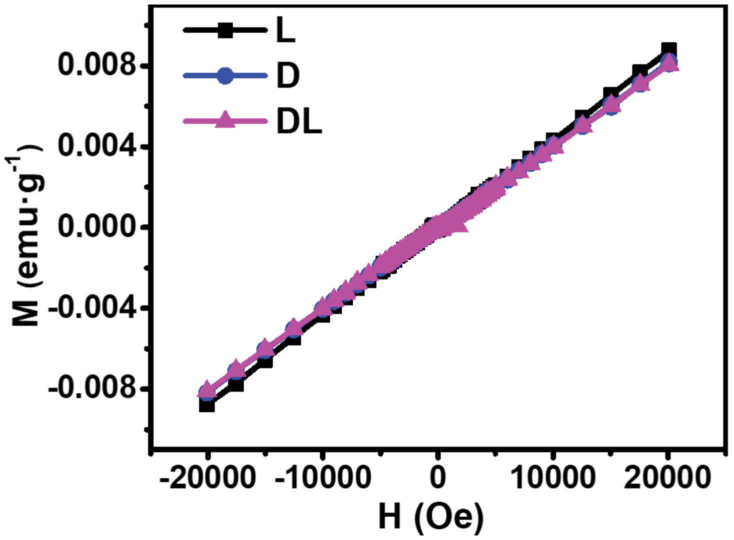

Figure S15. Magnetization loops of L-ZnO, D-ZnO and DL-ZnO.

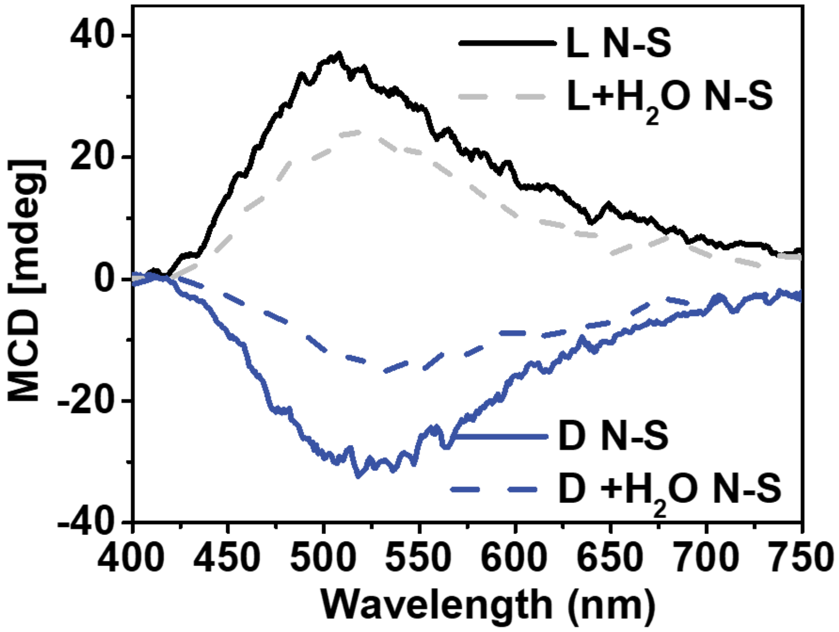

Figure S16. MCD spectra of dried films (solid lines) and films infiltrated with saturated water (dashed lines) under parallel applied magnetic fields of L-ZnO and D-ZnO.

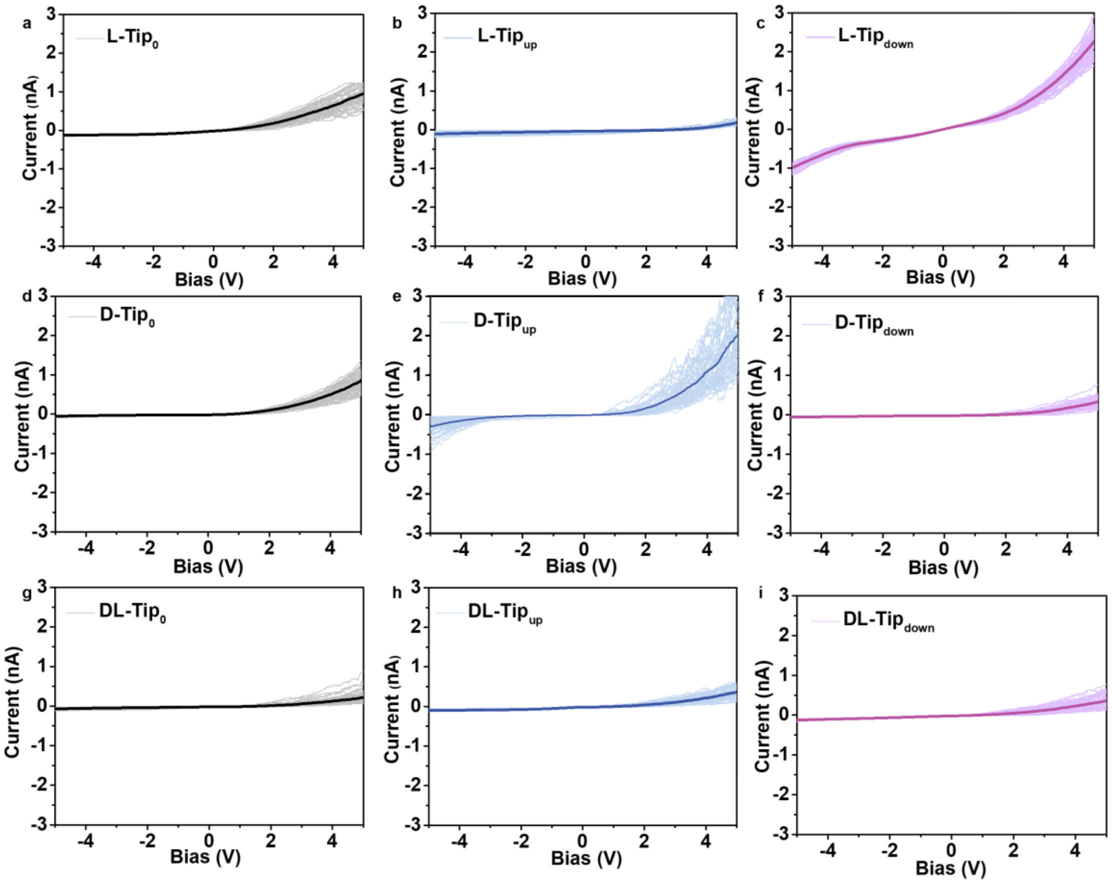

Figure S17. I-V curves obtained from L-ZnO, D-ZnO and DL-ZnO using mc-AFM. (a-c) The current as a function of the applied voltage I-V curves of L-ZnO with the tips magnetized in the nonmagnetized (black), up (blue) and down (purple). (d-f) The current as a function of the applied voltage I-V curves of D-ZnO with the tips magnetized in the nonmagnetized (black), up (blue) and down (purple). (g-i) The current as a function of the applied voltage I-V curves of DL-ZnO with the tips magnetized in the nonmagnetized (black), up (blue) and down (purple).

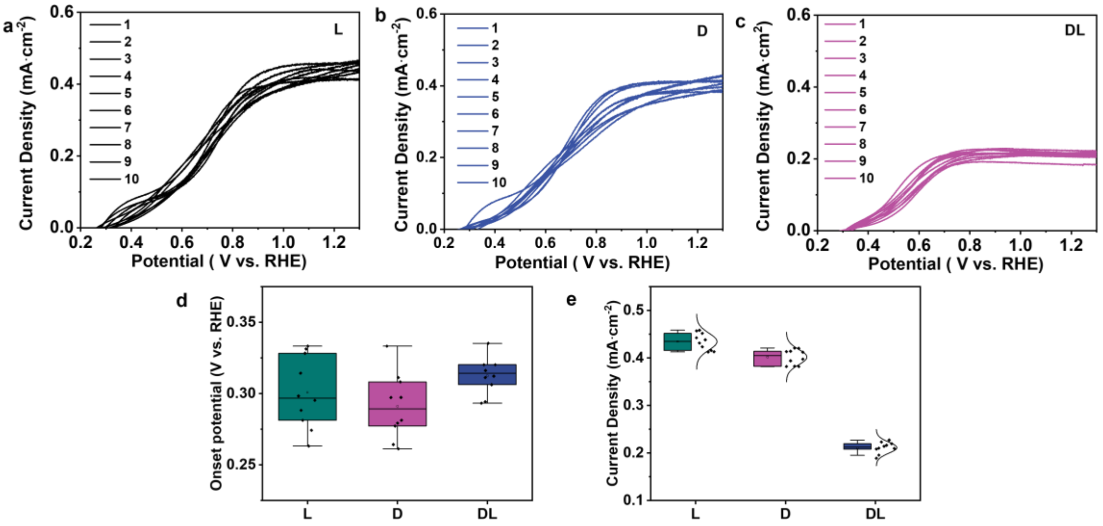

Figure S18. Reproducibility of PEC performance of (a) L-ZnO, (b) D-ZnO and (c) DL-ZnO. (d) Statistics of onset potential and (e) current density at 1.23 V vs. RHE of L-ZnO, D-ZnO and DL-ZnO.

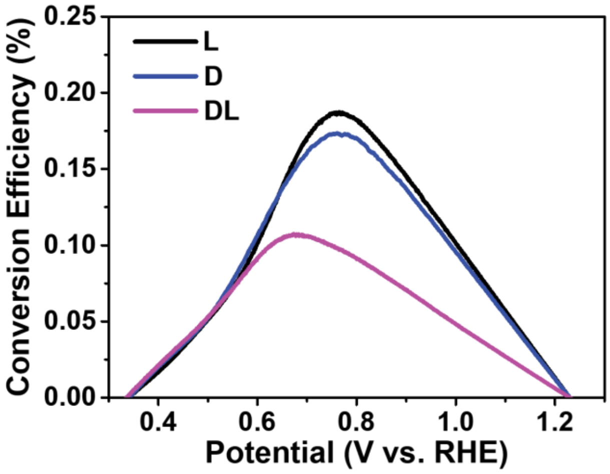

Figure S19. ABPE curves of L-ZnO, D-ZnO and DL-ZnO.

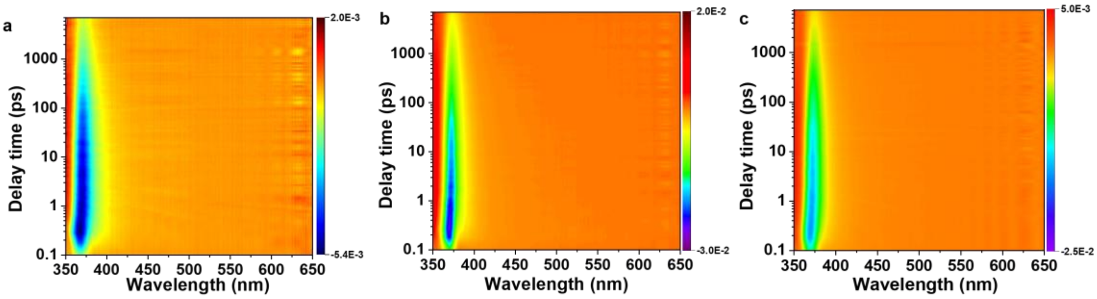

Figure S20. Pseudocolor transient absorption spectra of (a) L-ZnO, (b) D-ZnO and (c) DL-ZnO.

Table S1. Performance comparison of ZnO-based photoanodes in this work and previous literatures.

Photoanode	Modification strategy	Electrolyte	Photocurrent	Reference
L-ZnO	Chiral structure	0.5 M Na_2SO_4	0.43 mA/cm^2 at 1.23 V_{RHE}	This work
D-ZnO			0.40 mA/cm^2 at 1.23 V_{RHE}	
ZnO (002)	Facet engineering	0.5 M Na_2SO_4	0.35 ± 0.01 mA/cm^2 at 1.2 V_{RHE}	5
ZnO/g-C_xN_y (1.0)	Conformal coating	0.1 M KOH	~0.25 mA/cm^2 at 1.23 V_{RHE}	6
ZnO:Co@ZIF-8	Doping and functionalization	pH=13 alkaline solution	<0.16 mA/cm^2 at 1.33 V_{RHE}	7
15 nm SnS_2/b-ZnO NW	Heterojunction	0.35 M Na_2SO_3 + 0.25 M Na_2S	0.36 mA/cm^2 at 1.23 V_{RHE}	8
In doped ZnO	Element doping	0.1 M Na_2SO_4	0.42 mA/cm^2 at 1.23 V_{RHE}	9
Al doped ZnO	Element doping	0.5 M Na_2SO_4	0.31 mA/cm^2 at 1.23 V_{RHE}	10
C-ZnO NRs	Element doping	0.5 M Na_2SO_4	14.8 µA/cm^2 at 0.9 V_{RHE}	11
N-ZnO			13 µA/cm^2 at 0.9 V_{RHE}	
S-ZnO			6 µA/cm^2 at 0.9 V_{RHE}	
ZnO/ZnS	Heterostructure	0.5 M Na_2SO_3	0.073 mA/cm^2 at 0.78 V_{RHE}	12
ZnO@ZIF-8/67	Ternary hierarchical semiconductor	0.5 M Na_2SO_4	0.11 mA/cm^2 at 1.23 V_{RHE}	13
ZnO QDs/NRs	Homojunction	0.5 M Na_2SO_4	0.42 mA/cm^2 at 1.23 V_{RHE}	14
ZnO@N-CD@ZIF-8	Photosensitization	0.5 M Na_2SO_4	~0.30 mA/cm^2 at 0.73 V_{RHE}	15

Table S2. Fitted lifetimes and their percentages of L-ZnO, D-ZnO and DL-ZnO.

**Samples**	**Decay life times (ps)**			**Fractional contribution (%)**			**Average carrier lifetimes (ps)**
	τ_1	τ_2	τ_3	f_1	f_2	f_3	
L-ZnO	22.7 ±6.3	335.4±70.3	10060±1870	20.7	38.7	40.6	4218.9
D-ZnO	1.4±0.4	126.0±27.5	5720±899	31.4	31.3	37.3	2173.5
DL-ZnO	0.53±0.3	113.0±24.8	5519±1140	35.8	32.9	31.3	1764.8

Supplementary References

1. Wang, D. *et al.* Low-cost synthesis of a nanocomposite of MoS_2 and alkali-activated halloysite nanotubes for photocatalytic RhB degradation. *CrystEngComm* **24**, 6498-6504 (2022).
2. Liu, Y. *et al.* Synergistic effects of g-C_3N_4 three-dimensional inverse opals and Ag modification toward high-efficiency photocatalytic H_2 evolution. *J. Clean. Prod.* **328**, 129745 (2021).
3. Hao, M. *et al.* Higher photocatalytic removal of organic pollutants using pangolin-like composites made of 3–4 atomic layers of MoS_2 nanosheets deposited on tourmaline. *Environ. Chem. Lett.* **19**, 3573-3582 (2021).
4. Bai, T. *et al.* Spin Selectivity of Chiral Mesostructured Iron Oxides with Different Magnetisms. *Small*, 2104509 (2022).
5. Pawar, A. U. *et al.* Crystal facet engineering of ZnO photoanode for the higher water splitting efficiency with proton transferable nafion film. *Nano Energy* **20**, 156-167 (2016).
6. Hajduk, Š. *et al.* Conformal carbon nitride coating as an efficient hole extraction layer for ZnO nanowires-based photoelectrochemical cells. *Adv. Mater. Interfaces* **4**, 1700924 (2017).
7. Galan-Gonzalez, A. *et al.* Cobalt-doped ZnO nanorods coated with nanoscale metal-organic framework shells for water-splitting photoanodes. *ACS Appl. Nano Mater.* **3**, 7781-7788 (2020).
8. Bagal, I. V. *et al.* Investigation of charge carrier dynamics in beaded ZnO nanowire decorated with SnS_2/IrO_x cocatalysts for enhanced photoelectrochemical water splitting. *Appl. Surf. Sci.* **613**, 156091 (2023).
9. Le, N. *et al.* Enhanced light absorption and charge separation of In-doped ZnO nanorod arrays for photoelectrochemical water-splitting application. *Int. J. Energ. Res.* **46**, 6264-6276 (2021).
10. Kant, R. *et al.* Fabrication of ZnO nanostructures using Al doped ZnO (AZO) templates for application in photoelectrochemical water splitting. *Appl. Surf. Sci.* **447**, 200-212 (2018).
11. Karmakar, K. *et al.* Stable and enhanced visible-light water electrolysis using C, N, and S surface functionalized ZnO nanorod photoanodes: engineering the absorption and electronic structure. *ACS Sustainable Chem. Eng.* **4**, 5693-5702 (2016).
12. Li, C. *et al.* ZnO/ZnS heterostructures grown on Zn foil substrate by hydrothermal method for photoelectrochemical water splitting. *Int. J. Hydrogen Energy* **44**, 25416-25427 (2019).
13. Jia, G. *et al.* 1D alignment of ZnO@ZIF-8/67 nanorod arrays for visible-light-driven photoelectrochemical water splitting. *Appl. Surf. Sci.* **448**, 254-260 (2018).
14. Chen, Y.-C. *et al.* Overall photoelectrochemical water splitting at low applied potential over ZnO quantum dots/nanorods homojunction. *Chem. Eng. J.* **368**, 746-753 (2019).
15. Han, H. *et al.* Highly ordered N-doped carbon dots photosensitizer on metal-organic framework-decorated ZnO nanotubes for improved photoelectrochemical water splitting. *Small* **15**, 1902771 (2019).
